# Digital financial inclusion and welfare: Effect, mechanism and imbalance

**DOI:** 10.1371/journal.pone.0278956

**Published:** 2023-12-11

**Authors:** YanLong Li, Jin Peng

**Affiliations:** 1 School of Economics, University of Chinese Academy of Social Sciences, Beijing, Beijing, China; 2 School of Politics and Public Administration, Zhengzhou University, Zhengzhou, Henan, China; Wuhan University, CHINA

## Abstract

This study utilizes provincial panel data from China spanning from 2013 to 2019 and considers indices such as residents’ income, employment, housing, education, and medical care to comprehensively measure the welfare index of each province using the coefficient of variation. It also examines the impact and mechanism of digital financial inclusion on welfare and evaluates its influence on welfare imbalance through panel data models and RIF regression. The estimation results of digital financial inclusion on welfare indicate that digital financial inclusion is an important driver elevating the income, employment, housing, education, medical treatment and other welfares of Chinese residents, and the conclusion remains robust after using instrumental variable estimation and replacing the measurement method of welfare. Regional differences reveal that digital financial inclusion has a greater contribution to the welfare in the Midwest. The effect mechanism test shows that digital financial inclusion improves the welfare by creating employment opportunities and plays a greater role in the Midwest, and will also improve the welfare by increasing the welfare expenditure. However, this mechanism will exacerbate the welfare imbalance between the Midwest and East. The estimation results of digital financial inclusion on the welfare imbalance show that digital financial inclusion reduces welfare imbalance between the central and western regions and the eastern regions, as well as within the central and western regions.

## 1. Introduction

For a long time, factors such as unequal development opportunities in different regions have led to welfare imbalance among regions. What we are now confronted is the contradiction between unbalanced and inadequate development and the people’s ever-growing needs for a better life in China. Issues most closely related to people’s lives include employment, education and housing, etc. It is critical to coordinate the education, income distribution, employment, medical treatment, housing and other aspects. Therefore, one of the main tasks of Chinese government at this stage is to improve the welfare of Chinese residents and promote balanced regional development.

With the advancement of digital technology, application powered by digital technology has become increasingly prevalent in the financial sector, and China has already become a global leader in the development of digital finance (Fungáčová and Weill, 2015; Tsai 2017) [1, 2]. According to the FinTech Report 2021-Digital Payments released by Statista, China has become the world’s largest digital payment market in 2020, with a digital payment amount of USD 2,496.5 billion, accounting for 45.6%, followed by the United States, with a digital payment amount of USD 1,035.4 billion, accounting for 18.915%. Due to its inclusiveness (Saima and pais, 2011; Gabor & Brooks, 2017) [3, 4], digital finance plays a crucial role in promoting the welfare and reducing unbalanced development (Park and Mercado, 2015) [5].

For example, due to its inclusiveness (Bold, et al, 2012) [6], digital financial inclusion can promote the employment of low-income families (Pearce, 2011) [7]. In reality, there are still many people who are disqualified for traditional financial services (Demirgüç-Kunt, Asli, and Leora F. Klapper. 2012) [8]. Digital finance can make financial services more accessible to groups that are disqualified for traditional financial services (Dev. SM, 2006) [9], and low-income residents can benefit from the development of digital finance by having more opportunities to apply for loans (Fonté, 2012) [10] to invest in education and entrepreneurship (Banerjee & Newman, 1993; Xie Xuanli et al., 2018; Zhang Xun et al., 2019) [11–13]. Moreover, digital finance improves the efficiency in government expenditures, reduces administrative costs, and increases taxes (Ali, 2019) [14], so that the government has more financial resources, thereby increasing the welfare expenditures in education, healthcare, employment, housing, and other aspects (Demirguc-Kunt et al 2017; Ozili PK 2021) [15, 16]. Therefore, digital financial inclusion is expected to narrow the gap in China’s income and other welfares.

Some scholars also contend that digital financial inclusion exerts a negative effect on relative poverty and generates greater financial risks. Affected by the popularity of digital infrastructure, the use of smart mobile devices, and personal digital literacy, digital finance cannot guarantee poor individuals access to low-interest loans to improve their living standards and productivity (Mader, 2018) [17]. However, the prevalence of digital financial inclusion increases the participation of credit market, and expanded access to credit market increases household consumption by changing the marginal propensity to consumption. However, easier access to credit market also increases the risk of households falling into debt traps (Yue et al, 2022) [18]. Compared with wealthier groups in society, the poor are exposed to higher costs of financial transactions when using financial products, and new risks such as service interruption, digital technology risks, cyber fraud, and information theft bring more serious negative effects to the poor, thereby reducing their social welfares (Ozili, 2020) [19]. To sum up, the effect of digital financial inclusion on China’s welfare and its imbalance needs to be further studied.

The objective of this paper is to explore the effect and mechanism of digital financial inclusion affecting the welfare, as well as the role of digital financial inclusion in the imbalance of regional welfare. Firstly, this paper selects 5 indexes of residents’ income, employment, housing, education and medical treatment, uses the coefficient of variation method to comprehensively measure the welfare indexes of each province in China, and evaluates the effect of digital financial inclusion on the welfare. Secondly, it mainly investigates the regional differences in the effect of digital financial inclusion on the East and Midwest and its effect mechanism. Thirdly, this paper examines the effect of digital financial inclusion on the imbalance of regional welfare by quantile regression, so as to comprehensively demonstrate the effect and mechanism of digital financial inclusion on welfare.

The contributions of this paper lie in relevant empirical evidences and enlightenments for improving China’s welfare and reducing the welfare imbalance by studying the effect and mechanism of digital financial inclusion on welfare. Firstly, while digital finance is an important factor affecting China’s welfare and the inclusiveness of digital finance has been studied in most existing studies from the perspectives of employment, entrepreneurship and income, the housing, medical treatment, education and other factors of welfare are not fully investigated. This paper comprehensively examines the effect of digital financial inclusion on welfare from five perspectives of income, employment, housing, education, and medical treatment. Secondly, although the development of digital finance has affected welfare, the transmission mechanism of digital finance affecting welfare is not fully studied. This paper analyzes the theoretical mechanism of digital finance affecting China’s welfare, and provides empirical evidence to support the transmission mechanism of digital financial inclusion affecting welfare in the empirical study. Finally, China still faces many new challenges in the new stage of development, and the problem of unbalanced and insufficient development remains prominent. This paper examines the effect of digital financial inclusion on China’s welfare imbalance by quantile regression.

This paper is structured as follows. Part 2 presents the theoretical mechanism and related literature on the effect of digital financial inclusion on welfare. Part 3 describes the research strategies, including the data, variables and models used in this paper. Part 4 elaborates on the main empirical results of this paper. Part 5 empirically tests the transmission mechanism of digital financial inclusion affecting welfare. Part 6 further examines the effect of digital financial inclusion on the welfare imbalance. Part 7 is the conclusions of this paper.

## 2. Theoretical mechanism and literature review

**Fig 1:**
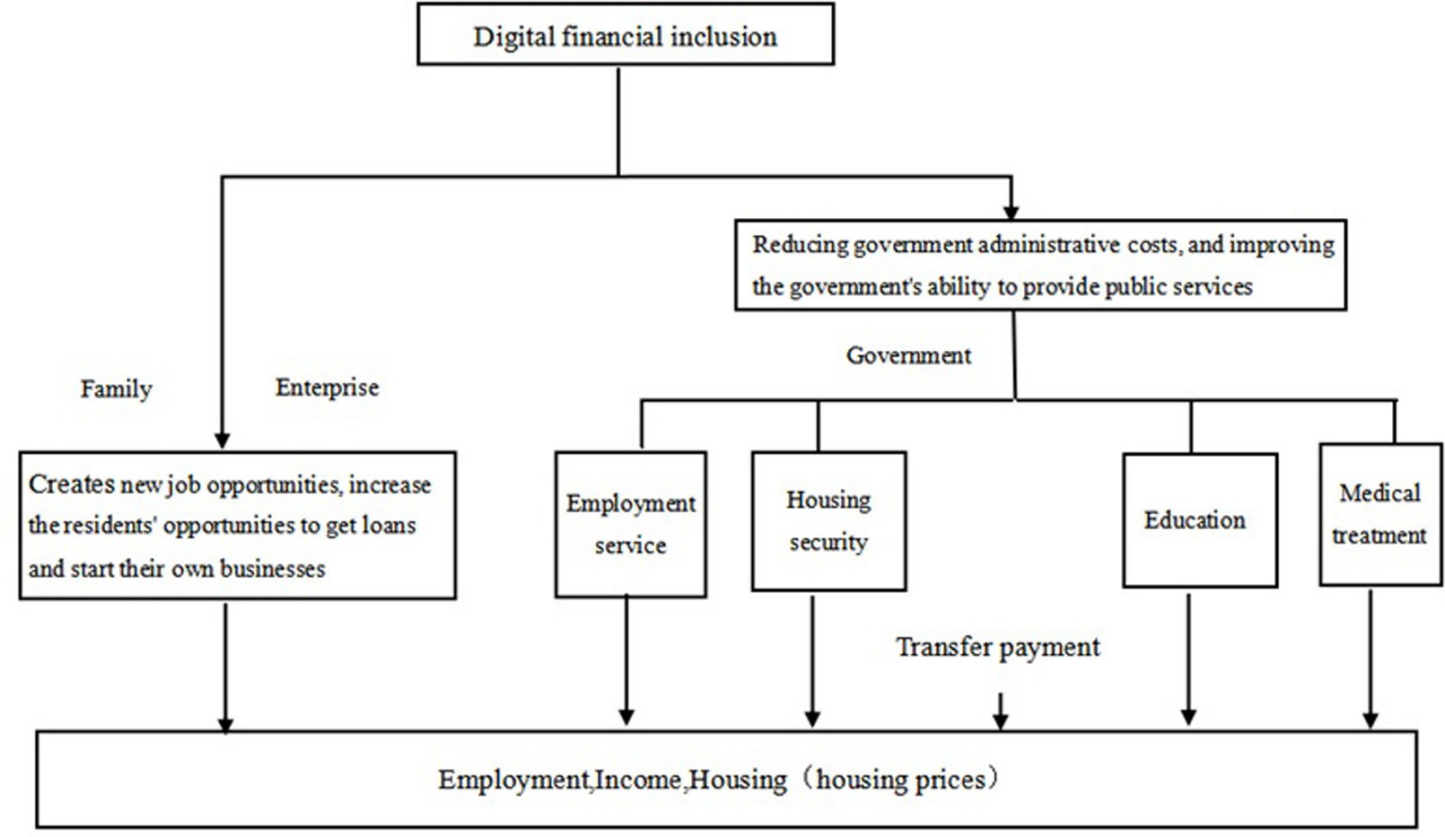
Theoretical framework of digital financial inclusion affecting welfare.

Digital financial inclusion refers to the extent to which digital finance is accessible and utilized. This paper works out the digital financial inclusion index through three dimensions: coverage, utilization, and digitalization. In China, the report of the 20th Party Congress has attached great importance to enhancing people’s welfare. To this end, a series of major measures have been deployed to improve the distribution system, implement pro-employment policies and perfect the social security system. The welfare in the report mainly includes income distribution, employment, medical care and social security, with social security primarily consisting of pension and housing. Consequently, improving income, employment, housing, education, and medical care, the five key components of welfare in the report, is almost certain to boost residents’ living standards. As an effective complement to traditional finance, the service scope and penetration of finance can be expanded by digital finance (Huang Yiping, 2016; Gabor & Brooks, 2017) [4, 20]. It is argued that digital financial inclusion can at least affect the welfare by promoting employment and entrepreneurship, reducing government administrative costs, and improving the government’s ability to provide public services, and has unique advantages in solving "unequal" development. The effect mechanism of digital financial inclusion on welfare is shown in Fig 1.

Firstly, digital financial inclusion creates new job opportunities, improve residents’ access to loans and entrepreneurial opportunities, and affects the residents’ employment, income and housing prices. Among them, benefited from more employment and entrepreneurial opportunities, digital financial inclusion can promote the welfare of residents in terms of employment and income. However, since digital finance enables more households and enterprises to have greater access to loans, it can transform the potential demand for house purchases into actual needs, and to a certain extent, it also drives up housing prices. Therefore, whether a significant boost to the housing welfare of residents is achieved depends on the relative extent to which digital finance acts on both incomes and housing prices. In addition to promoting welfare, digital financial inclusion also plays an important role in reducing the welfare imbalance due to its inclusiveness. For example, digital financial inclusion has boosted the scale of employment, including in producer services, consumer services and public organizations, thus providing more jobs. Those who could not find a job due to restrictions on knowledge, skills, education, etc., can enter the online car-hailing, takeaway and other industries after proper training, thereby promoting employment and income growth.

The transmission mechanism through which digital financial inclusion improves welfare by creating new jobs and entrepreneurial opportunities can also be empirically supported by copious literature. Digital financial inclusion provides funds for entrepreneurship, which can not only promote innovation and economic growth, but also create a large number of jobs (Baumol, 1968; King & Levine, 1993; Samila & Sorenson, 2011) [21–24]. Due to the imperfection of the financial market, poor households cannot borrow money to invest, which makes poverty alleviation more difficult (Banerjee & Newman, 1993) [11]. Dupas & Robinson (2013) find that market vendors (mainly women) in rural areas of Kenya can help poor households save more money [25]. Song Xiaoling (2017) finds through empirical analysis that digital financial inclusion lowers the threshold for accessing financial services, and helps to develop adaptable financial products and narrow the urban-rural income gap [26]. Aaron Alesane (2022) also discovers that digital financial inclusion can contribute to poverty reduction by studying the development of microfinance in Ghana [27].

Secondly, with the rapid development of digital financial inclusion, government’s ability to provide basic public services has been elevated, which will in turn improve the government’s welfare expenditure and transfer payment efficiency in employment, medical treatment, education, housing, etc., thus affecting the residents’ welfare in employment, medical treatment, education, housing, etc. For a long time, the government has been more willing to funnel financial resources in the production sector despite limited financial resources. Financial welfare expenditure accounts for a relatively low proportion, while the government productive expenditure also occupies a relatively high proportion in China’s financial expenditure structure. The development of digital finance such as digital payment reduces the administrative cost of the government, improves public service capabilities, and increases the efficiency in financial welfare expenditure, so it will increase the financial welfare expenditure. From another perspective, the probability of tax evasion increases significantly with income (Alstadsæter et al., 2019) [28], and the development of digital technologies helps reduce tax evasion, while increasing government revenue to facilitate the implementation of public policies.

Aker & Mbiti (2010) find that the development of digital financial inclusion can help improve tax collection and government services, lower administrative costs, reduce the “leakage” of funds during transfer, and strengthen the government’s ability to implement basic public services [29]. The capability of biological recognition systems such as face recognition improves the government’s performance in implementing public service projects (Gelb & Clark, 2013) [30]. During the COVID-19 pandemic, people not covered by digital financial inclusion services have become an obstacle for the government to implement public services. For example, since 7% of people in the United States do not have a bank account, the U.S. government is forced to pay by check. Emergency aids in Brazil have also been significantly disrupted, as 30% of people do not have a bank account. The pension industry will benefit from the development of fintech to address issues such as insufficient savings of plan members and formulate tailor-made retirement solutions (Terekhova, 2016) [31]. Through empirical study, Wang Yanan et al. (2021) find that digital financial inclusion has significantly improved social security through its effects on income and employment, and its promotional effect on medical insurance is superior to pension insurance and unemployment insurance [32]. Digital financial inclusion is also conducive to improving people’s well-being (Ganong and Noel, 2019) [33].

In general, the employment and income in welfare are considerably studied in existing literature, but the research on the effect and its mechanism of digital financial inclusion on welfare, especially on education, medical treatment, housing, etc. are scarce. This paper aims to comprehensively investigate the effect of digital financial inclusion on welfare from five aspects of income, employment, housing, education and medical treatment based on the Peking University Digital Financial Inclusion Index, verify the transmission mechanism of digital financial inclusion affecting the welfare, and empirically investigate the effect of digital financial inclusion on welfare imbalance. This paper attempts to arrive at a conclusion on whether the effect of digital financial inclusion development on welfare is dominated by "digital divide" or the expansion of the "digital divide" by studying the effect and mechanism of digital financial inclusion on welfare, thereby offering insights into how to improve welfare and reduce the welfare imbalance in China.

## 3. Research strategies

To fully demonstrate the effect and mechanism of digital financial inclusion affecting the welfare, the following research strategies are adopted. Based on some indexes of residents’ income, employment, housing, education, and medical treatment, the coefficient of variation method is used to comprehensively measure the welfare indexes of each province in China, and then evaluate the effect of digital financial inclusion on welfare. Through the above analysis, this paper examines the difference in the effect of digital financial inclusion on welfare between the East and Midwest and its effect mechanism. Finally, to further reflect the effect of digital financial inclusion on welfare imbalance, this paper investigates the effect of digital financial inclusion on regional differences in welfare by quantile regression. In this part, the data, variables, and models used in the above research strategies are introduced.

### 3.1. Data and variables

#### 3.1.1 Data source and description

The data used in this paper is the provincial panel data of 31 provinces and cities in China, with a time span from 2011 to 2019. The data on deposits and loans of financial institutions used in this paper is from the Almanac of China’s Finance and Banking, the number of employees in each province comes from the statistical yearbook of each province, the digital financial inclusion indexes for each province are from the Digital Finance Research Center of Peking University, and the remaining provincial-level data are sourced from the China Statistical Yearbook 2011–2019. Due to the lack of data before 2012 regarding some welfare indexes, the welfare data used in this paper to measure the effect of digital financial inclusion on the general welfare indexes is the data from 2013 to 2019, with a total of 217 samples.

#### 3.1.2 Dependent variables

The core dependent variables used in this paper are the welfare indexes of each province. Since income, employment, housing, education, and medical treatment are five important welfare indexes in China at this stage, this paper also calculates the people’s welfare indexes in China by drawing on the practices of Xu Xianchun et al. (2019) [34] accordingly. The specific variables selected are as follows: (1) Income variables include per capita disposable income and per capita consumption expenditure. To eliminate the effect of price factors, this paper converts it into the constant price in 2011 according to the consumer price index (CPI); (2) The employment variable is measured by the employment rate. The employment rate is calculated by dividing the number of employees by number of population aged 15 ∼ 64. The number of population aged 15 ∼ 64 is calculated according to the total population, dependency ratio and other data of each province; (3) The housing variable is measured by the housing price-to-income ratio, that is, the ratio of per capita disposable income to the average housing price. Although the boosting effect of digital financial inclusion on income is supported by a large number of literature, digital finance will also drive up housing prices with the increase of income. Therefore, the actual effect on the welfare is unknown in terms of housing; (4) The education welfare index is measured by the per capita education expenditure of high school students and below in each province (converted to the constant price in 2011 according to CPI), and the student-faculty ratio of high schools and below; (5) Medical welfare index is measured by the number of health technicians per 10,000 people and the number of beds in medical institutions per 10,000 people.

Before calculating the comprehensive welfare index accordingly, the data of each index need to be dimensionlessly processed. A commonly used dimensionless quantity method is the linear effect function method, of which the formula is as follows:

$$dx=(x-x_{\min})\;/\;(x_{\max}-x_{\min});$$ (1)

Where, x_min_ and x_max_ refer to the minimum and maximum values of the index x, respectively, and dx is the data after dimensionless processing of the index x. The dimensionless quantity method used in the calculation of the digital financial inclusion index is the logarithmic effect function method. To ensure that the core explanatory variables and core dependent variables are processed by the same dimensionless quantity method and the effect of extreme values is eliminated, the logarithmic effect function method is also used, of which the formula is as follows:

$$dx=(\text{ln}\;x-\text{ln}\;x_{\min})\;/\;(\text{ln}\;x_{\max}-\text{ln}\;x_{\min});$$ (2)

Among them, compared with the linear effect function method, the logarithmic effect function method can attenuate the effects of the outliers. In the following section, this paper will take the welfare indexes measured after the dimensionless processing of the logarithmic effect function method as the benchmark, and formula (1) is used, namely, the welfare index after the dimensionless processing of the linear effect function method, for robustness test.

After dimensionless processing, the weight of each index needs to be determined. This paper calculates the index as follows according to the coefficient of variation method among the objective weighting methods: 1. Calculate the coefficient of variation CV_j_ of the dimensionless index j (j = 1,2,⋯8, where the coefficient of variation (CV_j_) = standard deviation (S_j_) / mean (M_j_); 2. Calculate the weight w_j_ of each index, where w_j_ = CV_j_/ΣCV_j_. After the weight of each index is obtained, the calculated value of the welfare index is equal to Σjw_j_.

The descriptive statistics of the welfare index calculated in this paper are shown in Table 1, in which the division of the East and Midwest refers to the classification method adopted by the National Bureau of Statistics. Table 1 shows that the welfare index exhibits a clear upward trend, whether in China or in the East and Midwest regions, indicating that China’s welfare has improved significantly in recent years. The welfare in the East is better than that in the Midwest, but the welfare index in the Midwest rises faster, indicating that the gap between the Midwest and East is narrowing. Furthermore, both in China and in the East and Midwest regions, the standard deviation has shown a downward trend, indicating that with the improvement of China’s welfare, the welfare gap among provinces is also narrowing. This paper suggests that the development of digital financial inclusion is an important factor affecting the regional inequality of welfare. The effect of digital financial inclusion on the welfare will be systematically investigated in the following section.

**Table 1:** Estimated results of basic model.

	China		East		Midwest	
Year	Mean	Standard deviation	Mean	Standard deviation	Mean	Standard deviation
2013	0.372	0.122	0.440	0.133	0.329	0.095
2014	0.408	0.110	0.462	0.118	0.373	0.091
2015	0.445	0.099	0.491	0.110	0.415	0.082
2016	0.470	0.097	0.512	0.108	0.443	0.081
2017	0.497	0.093	0.538	0.109	0.471	0.074
2018	0.513	0.088	0.551	0.106	0.489	0.066
2019	0.535	0.084	0.570	0.105	0.512	0.061

#### 3.1.3 Explanatory variable

The core explanatory variable of this paper is the development level of digital financial inclusion (*index*, Guo Feng et al., 2020) [35]. The Peking University Digital Financial Inclusion Index of China (PKU_DFIIC) is compiled by the Institute of Digital Finance of Peking University and Ant Research. Based on the traditional financial inclusion indices proposed by existing literature and international organizations, coupled with the new developments and characteristics of digital financial services and the availability and reliability of data, the team constructed the digital financial inclusion index system through three dimensions: coverage, utilization and digitalization of financial inclusion. The current digital financial inclusion index contains the above three dimensions and 33 specific indices. In line with the above index system and the “analytic hierarchy process" commonly used in similar literature, the team finally compiled three-level PKU_DFIIC covering 31 provinces (municipalities directly under the central government and autonomous regions), 337 prefecture-level cities (prefectures, autonomous prefectures, leagues, etc.) and about 2,800 counties (county-level cities, banners, districts, etc.) in China’s mainland. The index is comparable both vertically and horizontally and more information thereof can be found at https://idf.pku.edu.cn/zsbz/515313.htm. For ease of interpretation, this paper has standardized the digital financial development index.

The control variables selected in this paper include: (1) Traditional financial development (*FD_trad*). Although the development of digital financial inclusion has a significant effect on the welfare, the effect of traditional financial development may also be remarkable. To more accurately determine the effect of digital financial inclusion on the welfare, the effect of traditional financial development needs to be eliminated. This paper determines the development level of traditional finance based on the ratio of the sum of deposits and loans in financial institutions to GDP; (2) Openness (*Trade*). Foreign trade has long been an important driver for economic growth, and exports can promote the improvement of the total factor productivity of local enterprises based on the "learning by export" effect (Zhang Jie et al., 2009) [36]. This paper determines the openness by the ratio of the total import and export of each province to GDP; (3) Dependency ratio (*Depen*). The dependency ratio may also be an important factor affecting China’s welfare. The higher the dependency ratio, the lower the proportion of the population aged 15–64, and the heavier the social burden, which will bring certain adverse effects on per capita income, per capita consumption expenditure, investments in education and medical treatment; (4) Economic structure (*Ind*). A region’s economic structure is another determinant of its welfare. For instance, the eastern provinces enjoy greater welfare as they have more industrial and commercial activities. This paper takes stock of the local economic structure using the proportion of value-added of the secondary and tertiary industries.

The regional variations and changes over time in the digital financial inclusion indices and control variables are shown in Table 2, from which it can be concluded that the digital financial inclusion indices of the eastern are greater than those of the central and western regions, with all regions showing an apparent upward trend. Traditional financial development, openness to the outside world, and the proportion of value-added of the secondary and tertiary industries, which manifest a pronounced uptrend over time, are also greater in the eastern regions. The dependency ratio variable is on a rising track over time, with the ratio in the central and western regions higher than that of the eastern regions.

**Table 2:** Regional differences and temporal trend changes of digital financial inclusion indices and explanatory variables.

	Eastern regions					Central and western regions				
	*index*	*FD_trad*	*trade*	*Depen*	*Ind*	*index*	*FD_trad*	*trade*	*Depen*	*Ind*
2012	-0.790	3.113	0.611	30.758	0.920	-1.179	2.507	0.127	36.853	0.879
2013	-0.101	3.185	0.579	31.683	0.922	-0.547	2.643	0.126	36.837	0.879
2014	0.142	3.247	0.544	32.550	0.926	-0.232	2.791	0.129	37.284	0.885
2015	0.620	3.610	0.464	33.650	0.927	0.238	3.078	0.107	38.226	0.885
2016	0.711	3.727	0.422	35.233	0.927	0.380	3.252	0.093	38.411	0.887
2017	1.231	3.711	0.436	36.117	0.935	0.849	3.341	0.100	39.937	0.896
2018	1.620	3.731	0.451	37.000	0.937	1.145	3.293	0.102	40.453	0.899

Finally, this paper summarized the basic information of each variable in Table 3.

**Table 3:** Variable definitions.

Variable	description	Measurement method	Data sources
*Welfare*	welfare indexes	Calculated from sub-dimensional indices	China Statistical Yearbook
*Index*	Digital financial inclusion index	Calculated from sub-dimensional indices	Digital Finance Research Center of Peking University
*FD_trad*	Financial development	the ratio of the sum of deposits and loans in financial institutions to GDP	Almanac of China’s Finance and Banking China Statistical Yearbook
*Trade*	Openness to the outside world平	the ratio of the total import and export of each province to GDP	China Statistical Yearbook
*Depen*	Dependency ratio	1-(Percentage of population aged 15∼64)	China Statistical Yearbook
*Ind*	Industrial structure	Valu-added of secondary and tertiary industries/GDP	China Statistical Yearbook

### 3.2 Model setting

The benchmark regression model used in the empirical analysis of this paper is as follows:

$$Wealfare_{it}=\lambda_{i}+\beta index_{it\text{-}1}+rX_{it\text{-}1}+u_{it};$$ (3)

Where the dependent variable Welfare is the comprehensive welfare index calculated by the coefficient of variation method, the core explanatory variable is the development level index of digital financial inclusion in each province and city, X is the control variable affecting the welfare, λ_i_ is the provincial fixed effect, and *u* is the random disturbance term. The effect of digital financial inclusion and explanatory variables on the welfare may have a certain time lag. In this paper, all explanatory variables are treated with a lag of one period, and the endogeneity problem that may be caused by bidirectional causality can be weakened. To investigate the effect of digital financial inclusion on various sub-indexes of the welfare, there is only a need to replace the dependent variable with each sub-index.

## 4. Empirical estimates of digital financial inclusion and welfare

Section 1 presents the benchmark regression results of how digital financial inclusion affects the welfare, Section 2 presents the robustness test results of this paper, Section 3 shows the regional differences in the effect of digital financial inclusion on the welfare, and Section 4 presents the estimation results of each sub-index of the welfare.

### 4.1 Digital financial inclusion and welfare: Benchmark regression

In this paper, the estimation is made based on model (3), and the estimation results are shown in Table 4. The estimation results in Table 4 show that when control variables such as traditional financial development, openness and dependency ratio are not controlled, the effect of digital financial inclusion on the welfare is positive and the estimated coefficient is significant at the 1% level. After the relevant control variables are controlled, the estimated coefficient of the digital financial inclusion index variable has decreased, but still positive and significant at the 1% level. This shows that digital financial inclusion has significantly increased China’s welfare index, and digital financial inclusion is an important driving factor for improving people’s welfare such as income, employment, housing, education, and medical treatment of Chinese residents.

**Table 4:** Digital financial inclusion and welfare: Benchmark regression.

	(1)	(2)
Dependent variables	Welfare index	Welfare index
*index*	0.071***	0.064***
	(36.56)	(17.36)
*FD_trad*		0.030***
		(5.02)
*Trade*		0.058***
		(2.42)
*Depen*		-0.001
		(-1.50)
*Ind*		0.278
		(1.39)
Sample size	217	217
*R* ^2^	0.878	0.896

*, **, *** indicate significant at 10%, 5%, and 1%, respectively, and the t-statistic values are given in brackets. Control variables include provincial fixed effects.

### 4.2 Robustness test

In this part, the robustness of the previous estimation results is tested. (1) The previous benchmark regression model may have endogeneity problems caused by missing variables and other reasons. The instrumental variable method is used for estimation. A common instrument variable is the Internet penetration rate inter (China Statistical Report on Internet Development). This paper selects the Internet penetration rate of each province as an instrument variable for robustness test. However, the Internet penetration rate may not be a perfect instrumental variable, and is likely to be directly related to the local infrastructure and welfare. Another common instrument variable is the distance from each province to Hangzhou. However, the distance from each province to Hangzhou does not change with time, so the panel data model of this paper cannot be directly estimated as an instrumental variable. In this regard, this paper selects the average development level of digital financial inclusion in neighboring provinces and cities as an instrumental variable, because digital finance has an obvious spatial agglomeration effect (Guo Feng et al., 2020), but the development of digital financial inclusion in adjacent provinces and cities will not directly affect the economic development of the province. This paper suggests that the average value of digital financial inclusion in neighboring provinces and cities is a reasonable instrumental variable [In light of the close economic ties between Hainan Province and Guangdong Province, the data is processed according to the practices of neighboring provinces.]. (2) Compared with the relative linear effect function method, the logarithmic effect function method can reduce the effect of the outliers. In this paper, the logarithmic effect function is used when the sub-indexes are dimensionlessly processed. However, the linear effect function method remains a method preferred in a great number of existing literature, and the logarithmic effect function method may not be a perfect dimensionless quantity method. The welfare index calculated by the coefficient of variation method after dimensionless processing of the linear effect function method is used as the dependent variable for robust estimation. (3) In addition to the dimensionless quantity method, there are also different methods for determining the weight of each index. In this paper, the weight of each index is calculated according to the coefficient of variation method. To ensure the robustness of the estimation results, this paper further adopts the entropy weight method to determine the weight of each index after dimensionless processing according to the logarithmic effect function, and then uses the comprehensive welfare index as the dependent variable for estimation.

The robustness test results of this paper are shown in Table 5. Among them, columns (1)-(2) present the first-stage estimation results of instrumental variables (with the Internet penetration rate and the average development level of digital financial inclusion in neighboring provinces and cities as instrumental variables), columns (3)-(4) present the corresponding estimation results of instrumental variables, column (5) shows the estimation results with the welfare indexes calculated after dimensionless processing of the linear effect function as the dependent variables, and column (6) shows the estimation results with the welfare indexes calculated after the index weight is determined by the entropy weight method as the dependent variables.

**Table 5:** Digital financial inclusion and welfare: Robustness test.

	(1)	(2)	(3)	(4)	(5)	(6)	(7)	(8)
Dependent variables	*index*	*index*	*index*	*index*	*Welfare*	*Welfare*	*Welfare*	*Welfare*
							Linear dimensionless	Weight of entropy weight method (EWM)
*index*				0.078***	0.064***	0.064***	0.054***	0.065***
				(15.67)	(17.29)	(17.30)	(19.17)	(17.78)
Internet penetration rate	10.525***		0.081					
	(19.05)		(0.44)					
index in neighboring provinces		0.997***	0.992***					
		(115.24)	(72.81)					
Sample size	248	217	217	217	217		217	217
LM statistic	128.24	214.08	214.09					
Wald-F statistic	261.52	13280.14	6610.57					
Sargan statistic			25.317					

*, **, *** indicate significant at 10%, 5%, and 1%, respectively, and the t-statistic values are given in brackets. Control variables include traditional financial development, openness, dependency ratio, economic structure and provincial fixed effects. The statistic of LM is Anderson canon. corr. LM statistic while the statistic of Wald-F is Cragg-Donald Wald F statistic. After adjusting the standard errors, Kleibergen-Paap rk LM statistic, Kleibergen- Paap rk Wald F statistic, and Hansen J statistic can be obtained. Nonetheless, the test results reveal no observable alteration.

The estimation results of the instrumental variables in columns (1)-(6) of Table 5 indicate that the Internet penetration rate and the development level of digital financial inclusion in neighboring provinces and cities are both important driving factors affecting the development of digital financial inclusion, the weak instrumental variable test and underidentification test is also passed. Whether the Internet penetration rate or the average value of digital financial inclusion in neighboring provinces and cities are used as instrumental variables, the estimation results still show that digital financial inclusion is an important driving factor for the improvement of welfare. However, the overidentification test statistic is greater than the critical value, thus ruling out the hypothesis that all instrumental variables are exogenous variables. This may be caused by the fact that Internet penetration is not a reasonable instrumental variable. The estimation results in columns (7)-(8) show that, regardless of whether dimensionless processing is done according to the linear effect function, or the welfare index calculated after the index weight is determined according to the entropy weight method is used as the dependent variable, the estimation results demonstrate that the estimated coefficient of the digital financial inclusion variable is positive and significant at the 1% level, which still shows that digital financial inclusion can significantly improve the welfare. In summary, the estimation results of this paper are relatively robust, and the core conclusions have high reliability.

### 4.3 Digital financial inclusion and welfare: Regional differences

This paper further investigates the regional differences in the effect of digital financial inclusion on welfare. According to the classification standard of the National Bureau of Statistics, each province and city is divided into the East and Midwest for estimation, and the differences in the effect of digital financial inclusion on the welfare between East and Midwest are compared, and the estimation results are shown in Table 6. The estimation results in columns (1)-(2) of Table 6 show that digital financial inclusion has a significant promotional effect on the welfare in both the East and Midwest, and the estimated coefficient of digital financial inclusion variable is larger in the Midwest. This shows that due to its inclusiveness, digital financial inclusion has a greater role in promoting the welfare in the Midwest, and is conducive to narrowing the gap in the welfare between Midwest and East, a "digital dividend" rather than a "digital divide" between Midwest and East. The estimation results in columns (3)-(6) show that even when the dependent variable is replaced by the welfare index calculated by the other two methods, it still shows that digital financial inclusion has a greater effect in the Midwest. This also indicates that no matter which measurement method is used, the core conclusions of this paper are consistent and the overall result is relatively robust. In the following analysis sections such as the effect mechanism, only the welfare index in the benchmark regression will be reported as the estimation result of the dependent variable(In fact,by replacing the dependent variables with the other two dependent variables, the core conclusions of the influence mechanism test are the same).

**Table 6:** Digital financial inclusion and welfare: Differences between East and Midwest.

Dependent variables	*Welfare*		*Welfare* (linear dimensionless)		*Welfare* (weight of entropy weight method) (EWM))	
Region	East	Midwest	East	Midwest	East	Midwest
	(1)	(2)	(3)	(4)	(5)	(6)
*index*	0.037***	0.075***	0.042***	0.059***	0.040***	0.076***
	(5.89)	(17.35)	(7.14)	(18.64)	(6.29)	(17.56)
Sample size	84	133	84	133	84	133
*R* ^2^	0.883	0.929	0.900	0.939	0.889	0.932

*, **, *** indicate significant at 10%, 5%, and 1%, respectively, and the t-statistic values are given in brackets. Control variables include traditional financial development, openness, dependency ratio, economic structure and provincial fixed effects.

### 4.4 Effect of digital financial inclusion on welfare sub-indexes

This paper further probes into the effect of digital financial inclusion on welfare indexes, and examines the effect of digital financial inclusion on the welfare. In this paper, the natural logarithm of per capita disposable income and per capita consumption expenditure, which reflects the income and welfare, the employment rate, which reflects residents’ employment welfare, the housing price-to-income ratio, which reflects residents’ housing, and the natural logarithm of per capita education expenditure for high school students and below, which reflects education welfare, respectively, student-faculty ratio of high school and below, the number of health technicians per 10,000 people, which reflects the medical welfare, and the number of beds in medical institutions per 10,000 people are used as dependent variables for estimation, and the difference of the effect of digital financial inclusion on each sub-index between East and Midwest is compared. The estimation results are shown in Table 7.

**Table 7:** Digital financial inclusion and welfare: Sub-indexes.

	Income		employment	housing	education		medical treatment	
	disposable income	consumption	employment rate	housing price-to-income ratio	educational expenditure	student-faculty ratio	technicians per 10,000 people	number of medical beds per 10,000 people
	(1)	(2)	(3)	(4)	(5)	(6)	(7)	(8)
China	0.163***	0.145***	0.009***	0.086*	0.157***	0.087***	6.824***	5.931***
	(34.49)	(25.29)	(2.83)	(1.80)	(14.57)	(2.85)	(9.37)	(19.21)
East	0.139***	0.124***	0.009	0.036	0.101***	-0.067	4.434**	3.821***
	(17.58)	(12.63)	(1.59)	(0.48)	(4.95)	(-1.14)	(2.17)	(7.72)
Midwest	0.172***	0.156***	0.009**	0.066	0.180***	0.161***	7.726***	6.924***
	(28.69)	(21.00)	(2.22)	(0.99)	(13.47)	(4.25)	(13.41)	(16.77)

*, **, *** indicate significant at 10%, 5%, and 1%, respectively, and the t-statistic values are given in brackets. Control variables include traditional financial development, openness, dependency ratio, economic structure and provincial fixed effects.

The estimation results in Table 7 show that digital financial inclusion has had a significant positive effect on income, employment, housing, education, and medical treatment, indicating that the development of digital financial inclusion positively affects various welfares. The estimation results by region show that, in terms of the effect of digital financial inclusion on welfare indexes, in addition to employment, digital financial inclusion has a greater effect in the Midwest. To sum up, digital financial inclusion plays a significant role in promoting each sub-index on the whole, which is generally greater in the Midwest. This also leads to the overall significant effect of digital financial inclusion on residents’ welfare, which is relatively greater in the Midwest, corresponding to the estimation results of the total index mentioned above.

## 5. Transmission mechanism of digital financial inclusion affecting welfare

This part further investigates the transmission mechanism through which digital financial inclusion affects the welfare. The previous analysis shows that, as the development of digital financial inclusion creates new employment opportunities, digital financial inclusion can promote the growth of residents’ employment, income and other welfares. However, while digital financial inclusion creates new jobs, these jobs tend to be in urban areas. Suri & Jack (2016) found that increased mobile payment penetration lifted 2% of Kenyan households out of poverty, and these benefits were partly driven by career choices (personal shifts from agriculture to business) [37]. Benefited from new employment opportunities, some households will reduce or even stop agricultural production and take up new jobs. Therefore, digital financial inclusion can encourage rural households to seek employment in urban areas, thus increasing the relative proportion of the urban population, which reflects more employment opportunities in urban areas. On this basis, this paper chooses the proportion of urban population to the total population (city), that is, the urbanization rate, as a proxy variable for employment opportunities and an intermediary variable of digital financial inclusion affecting the welfare.

In addition, digital financial inclusion can improve the welfare by elevating the efficiency of government administrative expenditure and the ability to implement public services. For a long time, productive expenditure has taken up a larger proportion in the government’s fiscal expenditure. Due to the high administrative cost and insufficient direct economic returns, the government’s expenditure on welfare is relatively low. Digital financial inclusion helps to reduce government administrative costs, improve the government’s ability to implement in the welfare sector, and the propensity to spend on welfare will increase. Therefore, this paper selects the fiscal expenditure in the welfare as an intermediary variable to test this mechanism. Considering that the welfare index covers income, employment, housing, education, medical care, etc., this paper takes the government’s education expenditure, employment and social security expenditure, medical health expenditure, and housing security expenditure as the financial welfare expenditure, and then converts it into the natural logarithm (Gov) of the constant price in 2011 according to the consumer price index, as another mediating variable for the effect of digital financial inclusion on the welfare. Through the above analysis, the econometric model estimated by this empirical test is as follows:

$$City_{it}=\alpha_{1i}+\beta_{1}index_{it\text{-}1}+r_{1}X_{it\text{-}1}+u_{1it};$$ (4)

$$Gov_{it}=\alpha_{2i}+\beta_{2}index_{it\text{-}1}+r_{2}X_{it\text{-}1}+u_{2it};$$ (5)

$$Welfare_{it}=\alpha_{3i}+\beta_{3}index_{it\text{-}1}+\beta_{4}City_{it-1}+r_{3}X_{it\text{-}1}+u_{3it};$$ (6)

$$Welfare_{it}=\alpha_{4i}+\beta_{5}index_{it\text{-}1}+\beta_{6}Gov_{it-1}+r_{4}X_{it\text{-}1}+u_{4it};$$ (7)

$$Welfare_{it}=\alpha_{5i}+\beta_{7}index_{it\text{-}1}+\beta_{8}City_{it-1}+\beta_{9}Gov_{it-1}+r_{5}X_{it\text{-}1}+u_{5it};$$ (8)

Models (4) and (5) are used to test the effect of digital financial inclusion on the transmission mechanism variables. Models (6) and (7) add the transmission benchmark variables based on the benchmark regression model (3) to test the effect of transmission mechanism variables on the welfare indexes. Model (8) is a model that includes two mediating variables at the same time. To reduce the possible endogeneity problem caused by bidirectional causality, the transmission mechanism variables in models (6)-(8) are treated with a one-period lag. This part first estimates models (4) and (5), and investigates the effect of digital financial inclusion on the urbanization rate and the proportion of welfare expenditures. The estimation results are shown in Table 8.

**Table 8:** Effect of digital financial inclusion on urbanization rate and expenditure on welfare.

	*City* (job opportunities)			*Gov*(Welfare expenditure)		
	China	East	Midwest	China	East	Midwest
	(1)	(2)	(3)	(4)	(5)	(6)
*index*	0.026***	0.016***	0.030***	0.144***	0.129***	0.153***
	(15.97)	(4.60)	(20.75)	(17.60)	(7.86)	(15.05)
*FD_trad*	0.013***	0.005	0.004	0.044***	0.056*	0.030
	(4.06)	(0.79)	(1.30)	(2.79)	(1.90)	(1.41)
*Trade*	0.106***	0.077***	-0.034	-0.316***	-0.253***	-0.561***
	(8.54)	(4.36)	(-1.33)	(-5.04)	(-3.11)	(-3.12)
*Depen*	-0.000	-0.000	0.001*	-0.002	0.003	-0.009**
	(-0.96)	(-0.55)	(1.68)	(-0.63)	(0.76)	(-2.30)
*Ind*	0.348***	1.197***	0.112	1.797***	3.699***	1.809***
	(3.43)	(4.41)	(1.40)	(3.51)	(2.94)	(3.21)
Sample size	248	96	152	248	96	152
*R* ^2^	0.851	0.720	0.943	0.898	0.915	0.895

*, **, *** indicate significant at 10%, 5%, and 1%, respectively, and the t-statistic values are given in brackets. Control variables include traditional financial development, openness, dependency ratio, economic structure and provincial fixed effects.

The estimation results in columns (1)-(3) of Table 8 show that digital financial inclusion has significantly promoted the urbanization rate, that is, employment opportunities in China, East and Midwest. The effect of digital financial inclusion on the urbanization rate is greater in Midwest than in the East. The estimation results in columns (4)-(6) show that digital financial inclusion has contributed to the increase of welfare expenditure, and the effect of digital financial inclusion on welfare expenditure is greater in Midwest than in the East. On the whole, the transmission mechanism of digital financial inclusion affecting the welfare efficiency by increasing employment opportunities and promoting the welfare expenditure has been preliminarily verified.

Although the estimation results in Table 7 show that digital financial inclusion can significantly affect employment opportunities and welfare expenditure, the transmission mechanism of digital financial inclusion affecting the welfare through job creation and welfare expenditure still needs to investigate the transmission mechanism variable, that is, the effect of intermediary variables on the welfare. This paper further estimates the models (6)-(8), and empirically tests the promotion effect of employment opportunities and welfare expenditure on the welfare, and the estimation results are shown in Table 9.

**Table 9:** Digital financial inclusion and welfare: Transmission mechanism test.

	*Welfare*	*Welfare*	*Welfare*	*Welfare*	*Welfare*	*Welfare*	*Welfare*	*Welfare*	*Welfare*
	China	East	Midwest	China	East	Midwest	China	East	Midwest
	(1)	(2)	(3)	(4)	(5)	(6)	(7)	(8)	(9)
*index*	0.050***	0.040***	0.049***	0.041***	0.010	0.058***	0.033***	0.013*	0.040***
	(9.73)	(5.77)	(5.46)	(8.06)	(1.47)	(9.49)	(5.86)	(1.94)	(4.49)
*City*	0.467***	-0.158	0.790***				0.331***	-0.285**	0.617***
	(3.65)	(-0.93)	(3.39)				(2.73)	(-2.09)	(2.67)
*Gov*				0.134***	0.168***	0.103***	0.122***	0.177***	0.085***
				(6.02)	(5.87)	(3.74)	(5.44)	(6.27)	(3.09)
Sample size	217	84	133	217	84	133	217	84	133
*R* ^2^	0.851	0.720	0.943	0.903	0.885	0.936	0.913	0.923	0.937

*, **, *** indicate sig*, **, *** indicate significant at 10%, 5%, and 1%, respectively, and the t-statistic values are given in brackets. Control variables include traditional financial development, openness, dependency ratio, economic structure and provincial fixed effects.

The estimation results in column (1) of Table 9 show that after adding the intermediary variable urbanization rate, the estimated coefficient of the digital financial inclusion variable is still significant at the 1% level, but has dropped significantly from 0.066 to 0.050 before adding the intermediary variable. The estimated coefficient of the mediating variable urbanization rate is positive and significant at the 1% level. This shows that the increase in employment opportunities has significantly promoted the improvement of welfare. Digital financial inclusion is an important transmission mechanism to improve residents’ employment, income and other welfare by creating employment opportunities. The estimated coefficients in columns (2) and (3) show that after adding the intermediary variable urbanization rate, the estimated coefficients of digital financial inclusion variables in the eastern and central and western regions change from 0.037 and 0.075 to 0.040 and 0.049, respectively. The estimated coefficients of the urbanization rate variable are -0.018 and 0.790 respectively, indicating that although digital financial inclusion is an important transmission mechanism to improve the welfare by creating employment opportunities, employment opportunities in Midwest have a greater effect on the welfare, and this transmission mechanism behaves more obviously in Midwest, that is, digital financial inclusion can promote the improvement of welfare in Midwest to a greater extent by creating employment opportunities, and can narrow the welfare gap between Midwest and East.

The estimation results in columns (4)-(6) of Table 9 show that the estimated coefficients of digital financial inclusion variables are still positive, and the estimated coefficients of the welfare expenditure variables are positive and pass significance test at the 1% level. According to the estimation results in Table 8, digital financial inclusion reduces administrative costs, increases administrative resources and welfare expenditures, which in turn promotes the improvement of welfare. However, welfare expenditures have a greater promotion effect. The transmission mechanism of digital financial inclusion to improve the welfare by increasing welfare expenditures will widen the gap between welfare in the Midwest and East. The possible reason is that the welfare such as education in the East is higher than that in the Midwest, and the welfare has a better effect.

In the end, the estimation results in columns (7)-(9) show that after incorporating these two transmission mechanism variables, the estimated coefficients are different from those in columns (1)-(6), but the estimation conclusion is basically consistent with the variables of the transmission mechanism. To sum up, digital financial inclusion has improved the welfare by creating new employment opportunities and promoting the welfare expenditure. Digital financial inclusion has a greater effect on welfare in Midwest by creating new employment opportunities. It is an important mechanism for the welfare gap between the Midwest and the East, but digital financial inclusion will widen the welfare gap between Midwest and East by increasing the welfare expenditure.

## 6. Further analysis: Effect of digital financial inclusion on the welfare imbalance

What we now face is the contradiction between unbalanced and inadequate development and the people’s ever-growing needs for a better life in China, and previous studies show that digital financial inclusion can reduce the welfare gap between the Midwest and East, that is, it helps to reduce the welfare imbalance between Midwest and East. However, the effect of digital financial inclusion on the welfare imbalance needs to be further verified. On the one hand, the division method of East and West is only one of the regional division standards, and different research conclusions may be drawn according to different division methods. On the other hand, has digital financial inclusion narrowed the welfare imbalance in the Midwest and East? Are there different effects on the welfare imbalance in the East and Midwest?

In this regard, this paper empirically tests the effect of digital financial inclusion on the welfare imbalance by RIF regression. The RIF regression equation is as follows:

$$v(F)=E_{y}(RIF(y;v))=\alpha_{5i}+\beta_{7}index_{it-1}+\lambda_{5}X_{it-1}+u_{5it};$$ (9)

The meaning of each explanatory variable is the same as in model (3), and *v* may be statistics that describe the distribution F(y), including mean, quantile, Gini coefficient and standard deviation. In this paper, y refers specifically to the welfare level of different regions, and u is the residual error of RIF regression. The statistics used in this paper to reflect the distribution of the welfare indexes are quantiles and standard deviations. The core conclusions drawn by using the variance and standard deviation are consistent, and this paper only gives the results when standard deviation is used as a statistic(The core conclusions drawn by using the variance and standard deviation are consistent, and this paper only gives the results when standard deviation is used as a statistic). The standard deviation can reflect the changes in the welfare gap as a whole, and different quantiles can describe the changes in the welfare level at each quantile, so as to obtain more detailed estimation results. This paper first estimates the national sample, and the estimation results are shown in Table 10.

**Table 10:** Digital financial inclusion and welfare: Transmission mechanism test.

Dependent variables	*Welfare*	*Welfare*	*Welfare*	*Welfare*
Quantile	0.25	0.5	0.75	Standard deviation
	(1)	(2)	(3)	(4)
*index*	0.090***	0.070***	0.026	-0.026***
	(3.47)	(4.61)	(1.49)	(-3.10)
Sample size	217	217	217	217
*R* ^2^	0.454	0.716	0.780	0.723

*, **, *** indicate significant at 10%, 5%, and 1%, respectively, and the t-statistic values are given in brackets. Control variables include traditional financial development, openness, dependency ratio, economic structure and provincial fixed effects.

The estimation results in columns (1)-(3) of Table 10 show that the estimated coefficient of the digital financial inclusion variable drops from 0.090 at the 0.25th quantile to 0.070 at the 0.5th quantile, and then further decreases to 0.026 at the 0.75th quantile, and the estimated coefficient at the 0.75th quantile does not pass the significance test. With the increase of the quantile, the estimated coefficients of digital financial inclusion variables show a clear downward trend, indicating that at the low welfare level, digital financial inclusion has a greater effect on promoting the welfare, which helps to narrow the welfare gap between regions. The estimation results in column (4) show that when the standard deviation is used as a statistic, the estimated coefficient of digital financial inclusion is negative and significant at the 1% level, which further shows that digital financial inclusion can significantly reduce the welfare imbalance in different regions.

It can be speculated that digital financial inclusion can also reduce the welfare imbalance in the East and Midwest, and this effect becomes more pronounced in the East. In this regard, this paper further divides the sample into the East and Midwest, and estimates the model (9). The estimation results are shown in Table 11.

**Table 11:** Imbalance between digital financial inclusion and welfare: Difference between East and Midwest.

	East				Midwest			
Quantile	0.25	0.5	0.75	Standard deviation	0.25	0.5	0.75	Standard deviation
	(1)	(2)	(3)	(4)	(5)	(6)	(7)	(8)
*index*	-0.027	0.066**	-0.023	0.010	0.083**	0.062***	0.053**	-0.026**
	(-0.62)	(2.30)	(-0.41)	(0.85)	(2.46)	(3.16)	(2.08)	(-2.18)
Sample size	84	84	84	84	133	133	133	133
*R* ^2^	0.534	0.802	0.762	0.816	0.450	0.678	0.653	0.459

*, **, *** indicate significant at 10%, 5%, and 1%, respectively, and the t-statistic values are given in brackets. The dependent variable is the people’s welfare index, and the control variables include traditional financial development, opening to the outside world, dependency ratio, economic structure and provincial fixed effects.

The estimation results in Table 11 show that the estimated coefficients of digital financial inclusion in the Midwest are greater than those in the East, indicating that digital financial inclusion can reduce the welfare imbalance between the Midwest and East, which is consistent with the previous conclusions. Moreover, digital financial inclusion reduces welfare imbalance between the Midwest and the East, as well as within the Midwest.

## 7. Conclusions, managerial implications and policy implications

This study utilizes provincial panel data from China spanning from 2013 to 2019 and considers indices such as residents’ income, employment, housing, education, and medical care to comprehensively measure the welfare index of each province using the coefficient of variation. It also examines the impact and mechanism of digital financial inclusion on welfare and evaluates its influence on welfare imbalance through panel data models and RIF regression. This paper offers a new research perspective on the effect of digital financial inclusion from the perspective of imbalance, and provides relevant empirical evidence and enlightenment for improving China’s welfare and reducing the welfare imbalance from the perspective of digital financial inclusion.

The major findings of this paper are elucidated as follows. Firstly, digital financial inclusion is an important driver improving Chinese residents’ income, employment, housing, education, medical treatment and other welfares. After carrying out instrumental variable estimation and replacing the welfare calculation method, the core conclusions remain the same. Secondly, digital financial inclusion has a greater role in promoting the welfare in the Midwest, and is conducive to narrowing the welfare imbalance between the Midwest and East, revealing a "digital dividend" rather than a "digital divide" between the Midwest and East. Thirdly, digital financial inclusion can improve the welfare by creating employment opportunities and to a greater extent, promote the welfare improvement in the Midwest, and reduce the welfare imbalance between the Midwest and East. Fourthly, digital financial inclusion reduces administrative costs, increases welfare expenditures, and promotes the improvement of welfare, but this effect is more remarkable in the East, and this transmission mechanism will increase the welfare imbalance in the Midwest and East. Fifthly, digital financial inclusion reduces welfare imbalance between the Midwest and the East, as well as within the Midwest.

We believe our study offers managerial implications in the following ways. First, welfare including residents’ income, employment, education, health care, and housing will improve significantly through digital financial inclusion. This study can help more people, particularly among groups such as the elderly, understand the importance of digital financial inclusion and its development, which, in turn, can maximize the positive impact. Second, digital financial inclusion can bridge the welfare gap between different regions and instill confidence in residents of underdeveloped areas about their future life. Therefore, it is essential for residents to acknowledge the inclusive and beneficial nature of digital financial inclusion, rather than seeing it as the reason for exacerbated welfare gap. Third, fintech companies should capitalize on the development opportunities in the digital era, which will not only promise rapid growth for the companies but also substantially contribute to the welfare of society, leading to a win-win outcome.

The above findings provide a useful reference for promoting the welfare improvement and reducing the welfare imbalance from the perspective of digital financial inclusion. Firstly, digital financial inclusion has boosted the welfare improvement and reduced the welfare imbalance. Therefore, it is still necessary to give full play to the positive role of digital financial inclusion, which can not only facilitate the welfare improvement in China, but also helps to reduce the welfare imbalance among regions. Secondly, the increase in employment opportunities has a greater effect on welfare in Midwest. Digital financial inclusion is an important effect mechanism to improve the welfare by creating employment opportunities. Therefore, to further deepen the development of digital financial inclusion and actively promote public employment services, turning potential employment demand into actual demand can further promote the improvement of China’s welfare. Thirdly, digital financial inclusion can improve the welfare by increasing the welfare expenditure, but this transmission mechanism is more effective in the East, which is not conducive to reducing the welfare imbalance in the Midwest and East. Therefore, we should increase the financial support for the welfare in the Midwest via policies to improve the welfare in the Midwest, and also need to improve the efficiency of welfare expenditure in the Midwest. Finally, digital financial inclusion reduces welfare imbalance between the Midwest and the East, as well as within the Midwest. Therefore, one of the development directions in the future is to further eliminate the “digital divide" especially in the Midwest and alleviate the welfare imbalance in the Midwest.

## 8. Limitations and future research

Although we have measured the impact of digital financial inclusion and welfare, there remain certain research limitations that can be further explored in the future. Firstly, limited by the availability of data, the research data in this paper are provincial panel data in China, but there are also great differences between Chinese cities, and the research in this paper has not delved into the city level. Secondly, although the welfare indexes constructed in this paper include income, employment, housing, education and medical treatment, subjective evaluation indexes such as utilisation of medical services per province, adult literacy rate per province, happiness are not yet included, so it may not be a perfect measurement index. One of the main research interests in the future is to include more detailed research data, such as city-level data or micro-resident survey data, find more indexes to measure the welfare, and study the effect of digital financial inclusion on welfare indexes (such as utilisation of medical services per province, adult literacy rate per province, residents’ happiness). Moreover, We will use micro-data to study the impact of digital inclusive finance on the welfare index and the heterogeneity between age and gender in the future.

## Supporting information

S1 Data (ZIP)

## References

[1] Fungáčová Z., & Weill L. Understanding financial inclusion in China. China Economic Review,2015, 34, 196–206.

[2] Tsai K. S. FinTech and Financial Inclusion in China. HKUST Institute for Emerging Market Studies,2017, No. 20.

[3] Sarma M, Pais J. Financial Inclusion and Developmen [J]. Journal of International Development, 2011, 23(5): 613–628.

[4] Gabor D, Brooks S. The digital revolution in financial inclusion: international development in the fintech era[J].New Political Economy,2017,22(4):1–14.

[5] Park C. Y, & Mercado R.. Financial inclusion, poverty, and income inequality in developing Asia. Asian Development Bank Economics Working Paper Series,2015, No. 426, 1–18.

[6] Bold C., Porteous D., & Rotman S., Social cash transfers and financial inclusion: Evidence from four countries. Consultative Group to Assist the Poor, 2012, No. 77, 1–28.

[7] Pearce D. Financial inclusion in the Middle East and North Africa: Analysis and roadmap recommendations. The World Bank, 2011.

[8] Demirgüç-Kunt A, Klapper L F. Financial inclusion in Africa: an overview [J]. World Bank Policy Research Working Paper, 2012 (6088).

[9] Dev S M. Financial inclusion: Issues and challenges[J]. Economic and political weekly, 2006: 4310–4313.

[10] Fonté E. F. Mobile Payments in the United States: How Disintermediation May Affect Delivery of Payment Functions, Financial Inclusion and Anti-Money Laundering Issues. Wash. JL Tech. & Arts, 2012:8, 419.

[11] Banerjee A V, Newman A F. Occupational choice and the process of development[J]. Journal of Political Economy, 1993, 101(2): 274–298.

[12] Xuanli Xie, Yan Shen, Haoxing Zhang, Feng Guo. Can digital finance promote entrepreneurship?-Evidence from China [J]. China Economic Quarterly, 2018, 17(4):1557–1580.(in Chinese).

[13] Xun Zhang, Guanghua Wan, Jiajia Zhang, Zongyue He. Digital Economy, Inclusive Finance and Inclusive Growth [J]. Economic Research, 2019, 54(8): 71–86.(in Chinese).

[14] Ali A. E. E. S. Empowering Women through Financial Inclusion: Some Evidence from Comoros. International Journal of Asian Social Science, 2019,9(2): 256–270.

[15] Demirgüç-Kunt A, Klapper L, Singer D, et al. The global Findex database 2017: measuring financial inclusion and opportunities to expand access to and use of financial services[J]. The World Bank Economic Review, 2020, 34(Supplement_1): S2–S8.

[16] Ozili P K. Financial inclusion research around the world: A review[C]//Forum for social economics. Routledge, 2021, 50(4): 457–479.

[17] Mader P. Contesting Financial Inclusion[J].Development and change,2018,49(2):461–483.

[18] Yue P, Korkmaz A G, Yin Z, et al. The rise of digital finance: Financial inclusion or debt trap[J].Finance Research Letters,2022,47(1):413–422.

[19] Ozili P K. Contesting Digital Finance for the Poor[J].Digital Policy, Regulation and Governance,2020,22(2):135–151.

[20] Yiping Huang. Internet finance solves the pain point of inclusive finance [J]. Enterprise Observer, 2016, 4(5): 49–51.(in Chinese).

[21] Baumol W J. Entrepreneurship in Economic Theory[J]. American Economic Review,1968, 58(2): 64–71.

[22] Karaivanov A. Financial Constraints and Occupational Choice in Thai Villages[J]. Journal of Development Economics,2012,97(2): 201–220.

[23] Samila S O Sorenson. Venture Capital, Entrepreneurship, and Economic Growth[J]. Review of Economics &Statistics,2011,93: 338–349.

[24] King R G, Levine R. Finance and growth: Schumpeter might be right[J]. The quarterly journal of economics, 1993, 108(3): 717–737.

[25] Dupas P, Robinson J. Savings Constraints and Microenterprise Development: Evidence from a Field Experiment in Kenya[J]. American Economic Journal: Applied Economics, 2013, 5(1):163–192.

[26] Xiaoling Song. An empirical test of digital financial inclusion in narrowing urban-rural income gap. [J].Finance & Economics, 2017,6:14–25.(in Chinese).

[27] Alesane Aaron. Microfinance, Livelihoods and Poverty Reduction in Ghana[M]. Taylor and Francis, 2022

[28] Alstadsæter A, Johannesen N, Zucman G. Tax evasion and inequality[J]. American Economic Review, 2019, 109(6): 2073–2103.

[29] Aker J C, Mbiti I M. Mobile phones and economic development in Africa[J]. Journal of Economic Perspectives, 2010, 24(3): 207–32.

[30] Gelb A, Clark J. Identification for development: the biometrics revolution[J]. Center for Global Development Working Paper, 2013 (315).

[31] Terekhova M. Is fintech really revolutionising UK pension schemes?[J]. Pensions Expert, 2016.

[32] Yanan Wang, Zhuohong Tan, Lekai Zheng. Research on the Effect of Digital Finance Inclusion on Social Security [J]. Journal of Quantitative & Technical Economics, 2020, 37(7): 92–112.(in Chinese).

[33] Ganong P., and Noel P.“Consumer Spending During Unemployment: Positive and Normative Implications”,American Economic Review,2019.109(7):2383–2424.

[34] Xianchun Xu, Zhengxi Zheng, Zhongwen Zhang.——Based on the comprehensive analysis of "China Balanced Development Index of Tsinghua University" [J]. Managing World, 2019,35(05):15–28.(in Chinese).

[35] Feng Guo, Jingyi Wang, Fang Wang, Tao Kong, Xun Zhang, Zhiyun Cheng. Measuring China’s Digital Financial Inclusion: Index Compilation and Spatial Characteristics [J], China Economic Quarterly, 2020, 19(4): 1401–1418.(in Chinese).

[36] Jie Zhang, Yong Li, Zhibiao Liu. Does Export Promote Productivity of Chinese Enterprises?——Empirical Evidence from Chinese Local Manufacturing Enterprises:1999∼2003[J]. Management World, 2009, 4(12):11–26.(in Chinese).

[37] Suri T, Jack W. The long-run poverty and gender Effects of mobile money[J]. Science, 2016, 354(6317): 1288–1292. 27940873 10.1126/science.aah5309

